# 3-[(*R*)-1-Hy­droxy­butan-2-yl]-1,2,3-benzo­triazin-4(3*H*)-one

**DOI:** 10.1107/S1600536812043802

**Published:** 2012-10-31

**Authors:** Fernando Rocha-Alonzo, David Morales-Morales, Simón Hernández-Ortega, Reyna Reyes-Martínez, Miguel Parra-Hake

**Affiliations:** a Departamento de Ciencias Químico Bilógicas, Universidad de Sonora, Hermosillo, Sonora, 83000 México; b Instituto de Química, Universidad Nacional Autónoma de México, Circuito exterior, Ciudad Universitaria, México D.F., 04510 México; c Centro de Graduados e Investigación, Instituto Tecnológico de Tijuana, Tijuana, B.C., 22500 México

## Abstract

The crystal structure of the title compound, C_11_H_13_N_3_O_2_, is stabilized by O—H⋯O hydrogen bonds, which link the mol­ecules into chains along [100].

## Related literature

For biological and synthetic applications of benzo-1,2,3-triazinones, see: Caliendo *et al.* (1999 ▶); Zheng *et al.* (2005 ▶); Vaisburg *et al.* (2004 ▶); Chollet *et al.* (2002 ▶); Le Diguarher *et al.* (2003 ▶); Clark *et al.* (1995 ▶); Carpino *et al.* (2004 ▶); Janout *et al.* (2003 ▶); Gierasch *et al.* (2000 ▶). For structures of benzo-1,2,3-triazinones, see: Hjortås *et al.* (1973 ▶); Hunt *et al.* (1983 ▶); Reingruber *et al.* (2009 ▶). For bond-length data, see: Allen *et al.* (1987 ▶). For the synthesis, see: Gómez *et al.* (2005 ▶).
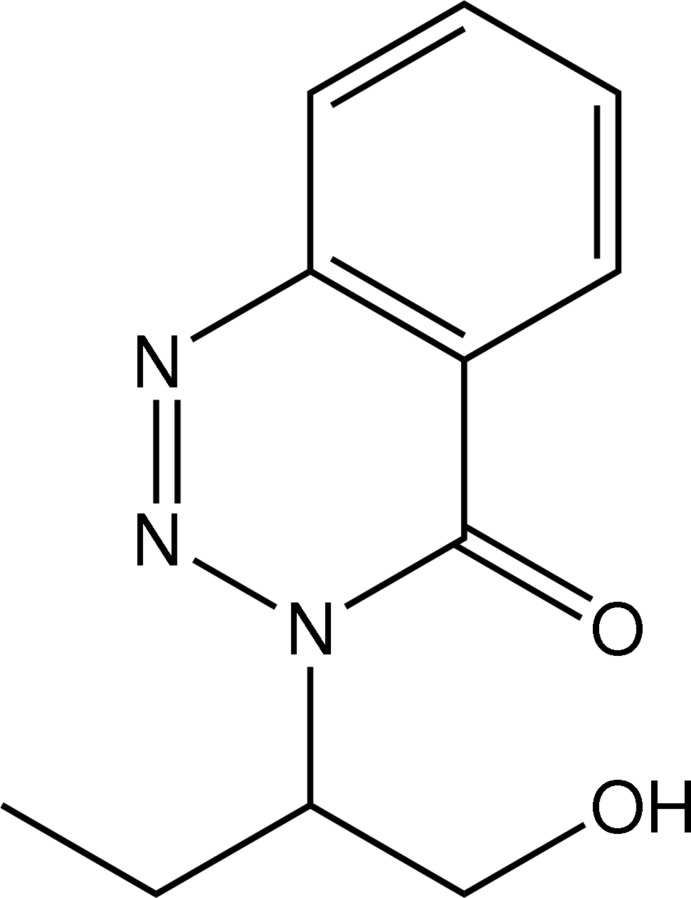


## Experimental

### 

#### Crystal data

C_11_H_13_N_3_O_2_
*M* *_r_* = 219.24
Orthorhombic, 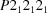

*a* = 8.9668 (13) Å
*b* = 10.1506 (15) Å
*c* = 12.0238 (17) Å
*V* = 1094.4 (3) Å^3^
*Z* = 4
Mo *K*α radiation
μ = 0.09 mm^−1^
*T* = 298 K
0.32 × 0.10 × 0.10 mm

#### Data collection

Bruker SMART APEX CCD area-detector diffractometer
9057 measured reflections
2000 independent reflections
1700 reflections with *I* > 2σ(*I*)
*R*_int_ = 0.044

#### Refinement

*R*[*F* ^2^ > 2σ(*F* ^2^)] = 0.034
*wR*(*F* ^2^) = 0.076
*S* = 0.93
2000 reflections
149 parameters
1 restraint
H atoms treated by a mixture of independent and constrained refinement
Δρ_max_ = 0.11 e Å^−3^
Δρ_min_ = −0.15 e Å^−3^

### 

Data collection: *SMART* (Bruker, 2007 ▶); cell refinement: *SAINT* (Bruker, 2007 ▶); data reduction: *SAINT*; program(s) used to solve structure: *SHELXTL* (Sheldrick, 2008 ▶); program(s) used to refine structure: *SHELXTL*; molecular graphics: *SHELXTL*; software used to prepare material for publication: *SHELXTL*.

## Supplementary Material

Crystal structure: contains datablock(s) I, global. DOI: 10.1107/S1600536812043802/zj2097sup1.cif

Structure factors: contains datablock(s) I. DOI: 10.1107/S1600536812043802/zj2097Isup2.hkl

Supplementary material file. DOI: 10.1107/S1600536812043802/zj2097Isup3.cml

Additional supplementary materials:  crystallographic information; 3D view; checkCIF report

## Figures and Tables

**Table 1:** Hydrogen-bond geometry (Å, °)

*D*—H⋯*A*	*D*—H	H⋯*A*	*D*⋯*A*	*D*—H⋯*A*
O2—H2⋯O1^i^	0.85 (1)	2.03 (1)	2.8712 (19)	171 (2)

Symmetry code: (i) 
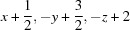
.

**Fig. 1.**
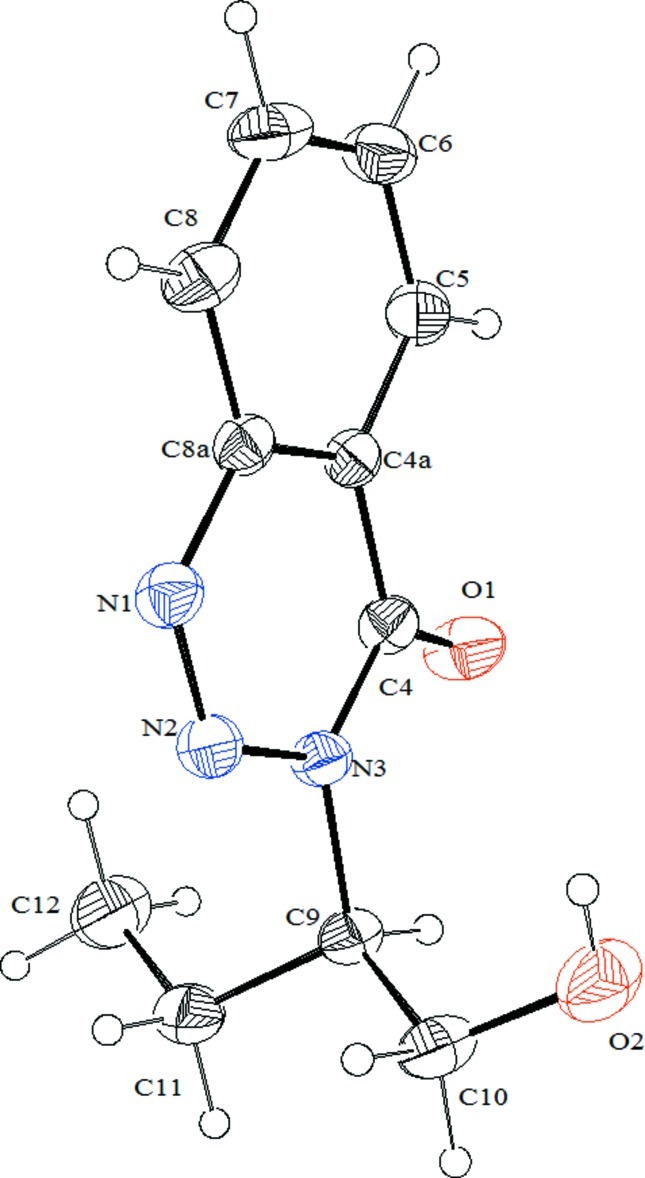
Molecular structure of (I) with displacement ellipsoids drawn at 50% probability.

**Fig. 2.**
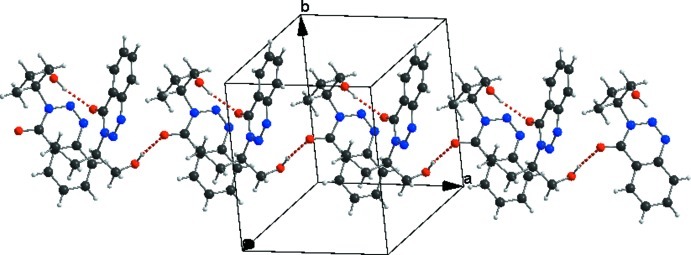
Packing of I showing the H-bonds. The molecules are forming a one-dimensional chain in the [100] direction. H-bonds are indicated by dashed lines.

## supplementary crystallographic information

### Comment

Benzo-1,2,3-triazinones are compounds widely investigated for their potential biological and chemical properties. These heterocyclic compounds have been studied as anesthetic (Caliendo *et al.*, 1999), anti-inflammatory (Zheng *et al.*, 2005), anticancer (Vaisburg *et al.*., 2004; Chollet *et al.*, 2002), and antitumoural (Le Diguarher *et al.*, 2003; Clark *et al.*, 1995) agents. In organic synthesis, 1,2,3-triazinones are used as an activating moiety in coupling agents for the preparation of peptides and amino acids (Carpino *et al.*, 2004; Janout *et al.*, 2003; Gierasch *et al.*, 2000). As result of its biological and synthetic importance, we have developed an alternative method for obtaining compounds with 1,2,3-triazinone moiety and in this paper we are describing the crystal structure of the title compound (I, Figure 1).

In the molecular structure of I, the N1=N2 bond [1.2636 (17) Å] is longer than the typical values for N=N double bonds (1.236 Å), whereas the N2–N3 bond [1.3735 (18) Å] is shorter than typical values for a N–N single bonds (1.404 Å) (Allen *et al.*., 1987). The structure of I shows co-planarity between two rings (1.30°). These measurements are in agreement with other benzo-1,2,3-triazinone crystal structure reports (Hjortås *et al.*, 1973; Hunt *et al.*, 1983; Reingruber *et al.*, 2009). Of interest to pharmaceutical applications, it has been suggested that co-planar structure in benzo-1,2,3-triazinones could give DNA-intercalating abilities such as those displayed by some anticancer agents (Reingruber *et al.*, 2009).

In the crystal structure, adjacent units are arranged into one-dimensional chain along [100] direction *via* O–H···O intermolecular hydrogen bonds (Figure 2 and Table 1).

### Experimental

The synthesis of the tittle compound included reagents and solvents of reagent grade, which were used without further purification. To a solution of 2-[(4*R*)-4-ethyl-4,5-dihydro-1,3-oxazol-2-yl]aniline (Gómez *et al.*, 2005) (0.89 g, 4.7 mmol, dissolved in 85 ml of methanol) was slowly added isoamyl nitrite (4.40 g, 37.6 mmol, 8 equiv) and the reaction mixture was stirred at room temperature until the disappearance of the aniline (followed by TLC, hexane/ethyl acetate, 3:1). The solvent was evaporated under reduced pressure to give a crude product that was purified by washing with petroleum ether and recrystallization from hexane/ethyl acetate. Crystalline colorless prisms of I were grown by slow diffusion of hexane over saturated ethyl acetate solutions of I. Yield > 99%, based on 2-[(4*R*)-4-Ethyl-4,5-dihydro-1,3-oxazol-2-yl]aniline; m.p., 89–90 °C. = -5.45° (*c* 0.22, MeOH). FTIR (KBr pellet, cm^-1^): 3439, 1686, 1663, 1296. ^1^H NMR [(CD_3_)_2_CO, 200 MHz] d 8.29 (ddd, *J* = 0.6, 1.5, 7.9 Hz, 2H), 8.16 (ddd, *J* = 0.6, 1.5, 8.1 Hz, 2H), 8.07 (ddd, *J* = 1.5, 7.0, 8.2 Hz, 2H), 7.91 (ddd,, *J* = 1.5, 7.0, 7.9 Hz, 2H), 5.22 (ddd, *J* = 5.1, 7.6, 15.5 Hz, 2H), 4.10 (dd, *J* = 8.4, 11.3 Hz, 2H), 3.96 (dd, J = 5.1, 11.3 Hz, 2H), 1.99 (dd, *J* = 7.5, 15.0 Hz, 4H), 0.90 (t, *J* = 7.4 Hz, 6H). ^13^C NMR [(CD_3_)_2_CO, 50 MHz] d 156.6, 144.5, 135.8, 133.2, 128.8, 125.7, 120.3, 64.2, 62.3, 24.2, 10.8. ESI-HRMS:220.1091 (100), calculated for [*M*+H]^+^, C_11_H_14_N_3_O_2_^+^, 220.1081; 192.1025 (8), calculated for [*M*+H—N_2_]^+^, C_11_H_14_NO_2_^+^, 192.1019. Anal for C_11_H_13_N_3_O_2_ (% Calcd./found) C, 60.26/60.73; H, 5.98/6.45; N, 19.17/19.47.

### Refinement

H atoms were included in calculated positions (C—H = 0.93 Å for aromatic H, C—H = 0.98 for methyn, C—H =0.97 Å for methylene H, and C—H= 0.96 Å for methyl H), and refined using a riding model, with *U*_iso_(H) = 1.2*U*_eq_ of the carrier atoms. The hydroxyl H atoms were located in a difference map and refined with O–H = 0.85±0.01 Å, and with *U*_iso_(H) = 1.2*U*_eq_(O).

### Crystal data

**Table:**

C_11_H_13_N_3_O_2_	*F*(000) = 464
*M**_r_* = 219.24	*D*_x_ = 1.331 Mg m^−^^3^
Orthorhombic, *P*2_1_2_1_2_1_	Mo *K*α radiation, λ = 0.71073 Å
Hall symbol: P 2ac 2ab	Cell parameters from 4276 reflections
*a* = 8.9668 (13) Å	θ = 2.6–25.2°
*b* = 10.1506 (15) Å	µ = 0.09 mm^−^^1^
*c* = 12.0238 (17) Å	*T* = 298 K
*V* = 1094.4 (3) Å^3^	Prism, colourless
*Z* = 4	0.32 × 0.10 × 0.10 mm

### Data collection

**Table:**

Bruker SMART APEX CCD area-detector diffractometer	1700 reflections with *I* > 2σ(*I*)
Radiation source: fine-focus sealed tube	*R*_int_ = 0.044
Graphite monochromator	θ_max_ = 25.4°, θ_min_ = 2.6°
Detector resolution: 0.83 pixels mm^-1^	*h* = −10→10
ω scans	*k* = −12→12
9057 measured reflections	*l* = −14→14
2000 independent reflections	

### Refinement

**Table:**

Refinement on *F*^2^	Primary atom site location: structure-invariant direct methods
Least-squares matrix: full	Secondary atom site location: difference Fourier map
*R*[*F*^2^ > 2σ(*F*^2^)] = 0.034	Hydrogen site location: inferred from neighbouring sites
*wR*(*F*^2^) = 0.076	H atoms treated by a mixture of independent and constrained refinement
*S* = 0.93	*w* = 1/[σ^2^(*F*_o_^2^) + (0.0412*P*)^2^] where *P* = (*F*_o_^2^ + 2*F*_c_^2^)/3
2000 reflections	(Δ/σ)_max_ < 0.001
149 parameters	Δρ_max_ = 0.11 e Å^−^^3^
1 restraint	Δρ_min_ = −0.15 e Å^−^^3^

### Special details

**Table:**

Geometry. All e.s.d.'s (except the e.s.d. in the dihedral angle between two l.s. planes) are estimated using the full covariance matrix. The cell e.s.d.'s are taken into account individually in the estimation of e.s.d.'s in distances, angles and torsion angles; correlations between e.s.d.'s in cell parameters are only used when they are defined by crystal symmetry. An approximate (isotropic) treatment of cell e.s.d.'s is used for estimating e.s.d.'s involving l.s. planes.
Refinement. Refinement of *F*^2^ against ALL reflections. The weighted *R*-factor *wR* and goodness of fit *S* are based on *F*^2^, conventional *R*-factors *R* are based on *F*, with *F* set to zero for negative *F*^2^. The threshold expression of *F*^2^ > σ(*F*^2^) is used only for calculating *R*-factors(gt) *etc*. and is not relevant to the choice of reflections for refinement. *R*-factors based on *F*^2^ are statistically about twice as large as those based on *F*, and *R*- factors based on ALL data will be even larger.

### Fractional atomic coordinates and isotropic or equivalent isotropic displacement parameters (Å^2^)

**Table:**

	*x*	*y*	*z*	*U*_iso_*/*U*_eq_	
O1	0.56004 (14)	0.68985 (11)	1.02583 (10)	0.0584 (4)	
N1	0.87770 (16)	0.64809 (13)	0.79106 (11)	0.0438 (4)	
O2	0.82337 (18)	0.99854 (13)	0.99816 (15)	0.0797 (5)	
H2	0.885 (2)	0.9362 (16)	0.992 (2)	0.096*	
N2	0.80536 (16)	0.75426 (13)	0.80106 (11)	0.0430 (4)	
N3	0.70312 (14)	0.76906 (12)	0.88520 (11)	0.0372 (3)	
C4	0.66320 (18)	0.67321 (15)	0.96026 (14)	0.0386 (4)	
C4A	0.75154 (18)	0.55429 (16)	0.95179 (13)	0.0362 (4)	
C5	0.7338 (2)	0.44941 (16)	1.02537 (14)	0.0469 (4)	
H5	0.6642	0.4546	1.0826	0.056*	
C6	0.8194 (2)	0.33848 (17)	1.01301 (16)	0.0545 (5)	
H6	0.8081	0.2686	1.0623	0.065*	
C7	0.9226 (2)	0.32979 (18)	0.92741 (17)	0.0568 (5)	
H7	0.9790	0.2535	0.9191	0.068*	
C8	0.94229 (19)	0.43244 (17)	0.85511 (16)	0.0517 (5)	
H8	1.0127	0.4266	0.7985	0.062*	
C8A	0.85625 (17)	0.54564 (15)	0.86684 (13)	0.0382 (4)	
C9	0.62783 (18)	0.89954 (14)	0.88408 (15)	0.0420 (4)	
H9	0.5594	0.9021	0.9476	0.050*	
C10	0.7406 (2)	1.00892 (17)	0.89991 (17)	0.0553 (5)	
H10A	0.8088	1.0088	0.8372	0.066*	
H10B	0.6886	1.0927	0.8998	0.066*	
C11	0.5352 (2)	0.91729 (17)	0.77929 (16)	0.0559 (5)	
H11A	0.4838	1.0012	0.7833	0.067*	
H11B	0.6015	0.9202	0.7156	0.067*	
C12	0.4222 (2)	0.80997 (19)	0.76146 (19)	0.0765 (7)	
H12A	0.3540	0.8081	0.8230	0.092*	
H12B	0.4722	0.7266	0.7560	0.092*	
H12C	0.3681	0.8266	0.6940	0.092*	

### Atomic displacement parameters (Å^2^)

**Table:**

	*U*^11^	*U*^22^	*U*^33^	*U*^12^	*U*^13^	*U*^23^
O1	0.0643 (8)	0.0449 (7)	0.0660 (9)	0.0042 (7)	0.0294 (8)	0.0035 (6)
N1	0.0471 (8)	0.0409 (8)	0.0434 (8)	0.0020 (7)	0.0101 (7)	−0.0012 (7)
O2	0.0754 (11)	0.0612 (10)	0.1026 (12)	0.0097 (8)	−0.0365 (10)	−0.0201 (9)
N2	0.0473 (8)	0.0401 (8)	0.0415 (8)	0.0014 (7)	0.0075 (7)	0.0013 (7)
N3	0.0410 (8)	0.0319 (7)	0.0387 (8)	0.0029 (6)	0.0047 (7)	0.0002 (6)
C4	0.0396 (9)	0.0364 (9)	0.0398 (9)	−0.0021 (8)	0.0043 (8)	−0.0018 (8)
C4A	0.0380 (9)	0.0339 (8)	0.0366 (9)	−0.0030 (7)	−0.0024 (8)	−0.0013 (7)
C5	0.0532 (11)	0.0426 (10)	0.0448 (10)	−0.0046 (9)	−0.0004 (9)	0.0019 (8)
C6	0.0638 (12)	0.0392 (10)	0.0604 (12)	−0.0009 (9)	−0.0109 (10)	0.0083 (9)
C7	0.0538 (12)	0.0365 (10)	0.0800 (14)	0.0100 (9)	−0.0071 (11)	−0.0013 (10)
C8	0.0435 (10)	0.0465 (11)	0.0651 (12)	0.0061 (9)	0.0060 (9)	−0.0089 (10)
C8A	0.0379 (9)	0.0352 (9)	0.0416 (10)	−0.0030 (7)	−0.0017 (8)	−0.0044 (8)
C9	0.0457 (9)	0.0337 (9)	0.0467 (10)	0.0058 (7)	0.0009 (9)	−0.0019 (8)
C10	0.0587 (11)	0.0362 (10)	0.0711 (12)	0.0034 (8)	−0.0022 (12)	−0.0055 (9)
C11	0.0648 (12)	0.0429 (10)	0.0600 (12)	0.0132 (9)	−0.0118 (10)	−0.0007 (9)
C12	0.0765 (14)	0.0602 (13)	0.0927 (17)	0.0111 (12)	−0.0357 (14)	−0.0133 (12)

### Geometric parameters (Å, º)

**Table:**

O1—C4	1.2271 (18)	C7—C8	1.368 (2)
N1—N2	1.2636 (17)	C7—H7	0.9300
N1—C8A	1.396 (2)	C8—C8A	1.391 (2)
O2—C10	1.399 (2)	C8—H8	0.9300
O2—H2	0.846 (9)	C9—C10	1.514 (2)
N2—N3	1.3735 (18)	C9—C11	1.520 (2)
N3—C4	1.3745 (19)	C9—H9	0.9800
N3—C9	1.4866 (19)	C10—H10A	0.9700
C4—C4A	1.447 (2)	C10—H10B	0.9700
C4A—C8A	1.390 (2)	C11—C12	1.503 (2)
C4A—C5	1.393 (2)	C11—H11A	0.9700
C5—C6	1.371 (2)	C11—H11B	0.9700
C5—H5	0.9300	C12—H12A	0.9600
C6—C7	1.387 (3)	C12—H12B	0.9600
C6—H6	0.9300	C12—H12C	0.9600
			
N2—N1—C8A	120.17 (13)	C8—C8A—N1	118.22 (15)
C10—O2—H2	109.5 (17)	N3—C9—C10	110.42 (13)
N1—N2—N3	120.40 (12)	N3—C9—C11	111.17 (14)
N2—N3—C4	125.45 (12)	C10—C9—C11	112.49 (14)
N2—N3—C9	113.20 (12)	N3—C9—H9	107.5
C4—N3—C9	121.22 (13)	C10—C9—H9	107.5
O1—C4—N3	121.38 (14)	C11—C9—H9	107.5
O1—C4—C4A	124.93 (15)	O2—C10—C9	113.91 (15)
N3—C4—C4A	113.68 (14)	O2—C10—H10A	108.8
C8A—C4A—C5	119.70 (15)	C9—C10—H10A	108.8
C8A—C4A—C4	118.29 (14)	O2—C10—H10B	108.8
C5—C4A—C4	122.01 (15)	C9—C10—H10B	108.8
C6—C5—C4A	119.66 (17)	H10A—C10—H10B	107.7
C6—C5—H5	120.2	C12—C11—C9	113.63 (15)
C4A—C5—H5	120.2	C12—C11—H11A	108.8
C5—C6—C7	120.42 (17)	C9—C11—H11A	108.8
C5—C6—H6	119.8	C12—C11—H11B	108.8
C7—C6—H6	119.8	C9—C11—H11B	108.8
C8—C7—C6	120.59 (17)	H11A—C11—H11B	107.7
C8—C7—H7	119.7	C11—C12—H12A	109.5
C6—C7—H7	119.7	C11—C12—H12B	109.5
C7—C8—C8A	119.55 (17)	H12A—C12—H12B	109.5
C7—C8—H8	120.2	C11—C12—H12C	109.5
C8A—C8—H8	120.2	H12A—C12—H12C	109.5
C4A—C8A—C8	120.07 (15)	H12B—C12—H12C	109.5
C4A—C8A—N1	121.71 (14)		

### Hydrogen-bond geometry (Å, º)

**Table:**

*D*—H···*A*	*D*—H	H···*A*	*D*···*A*	*D*—H···*A*
O2—H2···O1^i^	0.85 (1)	2.03 (1)	2.8712 (19)	171 (2)

Symmetry code: (i) *x*+1/2, −*y*+3/2, −*z*+2.
